# T_H_17 Cell Frequency in Peripheral Blood Is Elevated in Overweight Children without Chronic Inflammatory Diseases

**DOI:** 10.3389/fimmu.2017.01543

**Published:** 2017-11-16

**Authors:** Theresa Isabell Schindler, Johanna-Josophina Wagner, Sybelle Goedicke-Fritz, Tobias Rogosch, Verena Coccejus, Verena Laudenbach, Wilfried Nikolaizik, Christoph Härtel, Rolf Felix Maier, Sebastian Kerzel, Michael Zemlin

**Affiliations:** ^1^Children’s Hospital, Philipps University, Marburg, Germany; ^2^Department of Pediatrics, Helios-Klinikum Buch, Berlin, Germany; ^3^Department of General Pediatrics and Neonatology, University Children’s Hospital of Saarland, Homburg, Germany; ^4^Department of Pediatrics, University of Lübeck, Lübeck, Germany; ^5^Department of Pediatric Pneumology and Allergy, University Children’s Hospital Regensburg, Regensburg, Germany

**Keywords:** T_H_17 cells, IL-17A, receptor-related orphan receptor C, inflammation, overweight, children

## Abstract

**Background:**

The prevalence of obesity has dramatically increased in children in the last few decades and is associated with chronic inflammatory diseases. Fat tissue produces IL-6 and TNF-α, which are stimuli for T_H_17 cell differentiation. These cells are characterized by expression of the transcription factor receptor-related orphan receptor C (RORC) and by IL-17A production. In murine models, obesity has been linked with elevated T_H_17 cell frequencies. The aim of this study was to explore whether being overweight was associated with an elevated frequency of circulating T_H_17 cells or elevated messenger RNA (mRNA)-levels of IL-17A and RORC in children without chronic inflammatory diseases.

**Methods:**

We studied peripheral blood samples from 15 overweight and 50 non-overweight children without a history of autoimmune diseases, asthma, atopic dermatitis or allergic rhinoconjunctivitis. T_H_17 cells were quantified in Ionomycin stimulated peripheral blood mononuclear cells by flow cytometry using intracellular IL-17A staining. RORC- and IL-17A expressions were measured by real-time PCR.

**Results:**

We found significantly elevated T_H_ cell frequencies in overweight children compared then on-overweight controls with 34.7 ± 1.5% of CD3^+^CD4^+^ cells versus 25.4 ± 2.4% (mean ± SEM, *p* = 0.0023), respectively. Moreover, T_H_ cell frequencies correlated positively with body mass index (*r* = 0.42, *p* = 0.0005, respectively). The relative mRNA expression of RORC (*p* = 0.013) and IL-17A (*p* = 0.014) were upregulated in overweight compared to non-overweight children.

**Conclusion:**

Childhood obesity is an independent factor that is associated with an elevated frequency of circulating T_H_17 cells and higher expression of RORC- and IL-17A-mRNA after *in vitro* stimulation with Ionomycin. This might be due to the inflammatory activity of the fat tissue. Studies on T_H_17 immunity should not only be adjusted for acute and chronic inflammatory diseases but also for overweight.

## Introduction

Being overweight not only affects adults by causing chronic diseases including type 2 diabetes, arterial hypertension, or coronary heart disease, but is also an increasingly widespread health issue in childhood (1, 2). In 2010, 43 million children worldwide were overweight and this number is likely to increase to 60 million by 2020 (3). Obesity is a risk factor for allergic diseases like bronchial asthma and for various autoimmune diseases (4, 5). It contributes to a low-grade chronic inflammation with higher numbers of macrophages, mast cells, neutrophils, B- and T-cells in adipose tissue (6, 7), but the underlying pathogenesis and complex mutual interactions between immunity and metabolism are not yet completely understood.

T_H_17 cells play a role in autoimmune and allergic inflammation (8–10). T_H_17 cells are characterized by retinoic acid receptor-related orphan receptor C (RORC) expression (11, 12) and IL-17A production which attracts and activates neutrophil granulocytes (13). Physiologically, T_H_17 cells seem to be primarily important for clearance of pathogens, which cannot be fought effectively by T_H_1 nor T_H_2 cells—for example, *Klebsiella pneumoniae, Borrelia burgdorferi*, and helminths or fungi (14, 15). On the other hand, T_H_17 cells contribute to various autoimmune and allergic diseases, for example, inflammatory bowel diseases or bronchial asthma (16).

Furthermore, there is growing evidence from animal models that T_H_17 cells might also contribute to a low-grade chronically sustained inflammation in obesity. Interestingly, T_H_17 cells were elevated in the spleens of mice with a diet-induced obesity compared to normal weight mice (17). Likewise, more T_H_17 cells were detected in adipose tissue of obese compared to normal weight mice (18). Łuczyński et al. found more T_H_17 cells in peripheral blood samples of children with central obesity and diabetes mellitus (19). However, to our knowledge, none of the published studies on T_H_17 cells in overweight was controlled for potentially coinciding chronic inflammatory disease like bronchial asthma or autoimmunity. Thus, it remains unknown whether elevated T_H_17 cells in overweight individuals are directly linked with overweight or are a consequence of associated chronic inflammatory diseases (20). In this study, we wanted to test the hypothesis that the T_H_17 cell frequency is elevated in the peripheral blood of overweight children without allergic or autoimmune disease.

## Materials and Methods

The study was performed at the *Department of Pediatrics, Philipps University, Marburg*, and was approved by the local Ethics Committee. Written informed consent was obtained from parents and in older children additionally from themselves. Children admitted to the outpatient clinic because of adiposity or non-inflammatory conditions like headache, elective surgery, or abdominal pain were screened for eligibility. Children were excluded from our study when they were younger than 4 years or older than 17 years old, had asthma, atopic dermatitis, allergic rhinoconjunctivitis, or any autoimmune disease in their medical history. Symptoms of an acute infection during the previous 2 weeks led to exclusion as well as clinical symptoms of a current infection, current C-reactive protein elevation or leukocytosis. Being overweight was defined as a body mass index (BMI) ≥90th percentile, while children with a BMI < 90th percentile were included in our non-overweight control group. BMI percentiles were assessed with percentiles for German children published by Kromeyer-Hauschild et al. (21).

### Flow Cytometry

2.6 ml of venous blood were collected after informed consent during a routinely performed venipuncture or during routine placement of a peripheral venous catheter.

Peripheral blood mononuclear cells (PBMCs) were isolated by Ficoll-Hypaque gradient (PAA, Linz, Austria) and were stimulated in a 12-well plate in X-VIVO 15 (X-VIVO 15 w/o Gentamycin and Phenol Red, Lonza, Velvier, Belgium) with Ionomycin (Sigma Aldrich Chemie, Steinheim, Germany) for 4 h (37°C, 5% CO_2_). Brefeldin A (eBioscience, San Diego, CA, USA) was added after 3 h. After staining these cells with anti-CD3 (FITC) and anti-CD4 (PE) antibodies (R&D Systems, Minneapolis, MN, USA), the PBMCs were fixated, permeabilized, and stained intracellularly with anti-IL-17A antibody (APC) (Flow Cytometry Fixation and Permeabilization Buffer Kit I, R&D Systems, Minneapolis, MN, USA).

**Figure 1:**
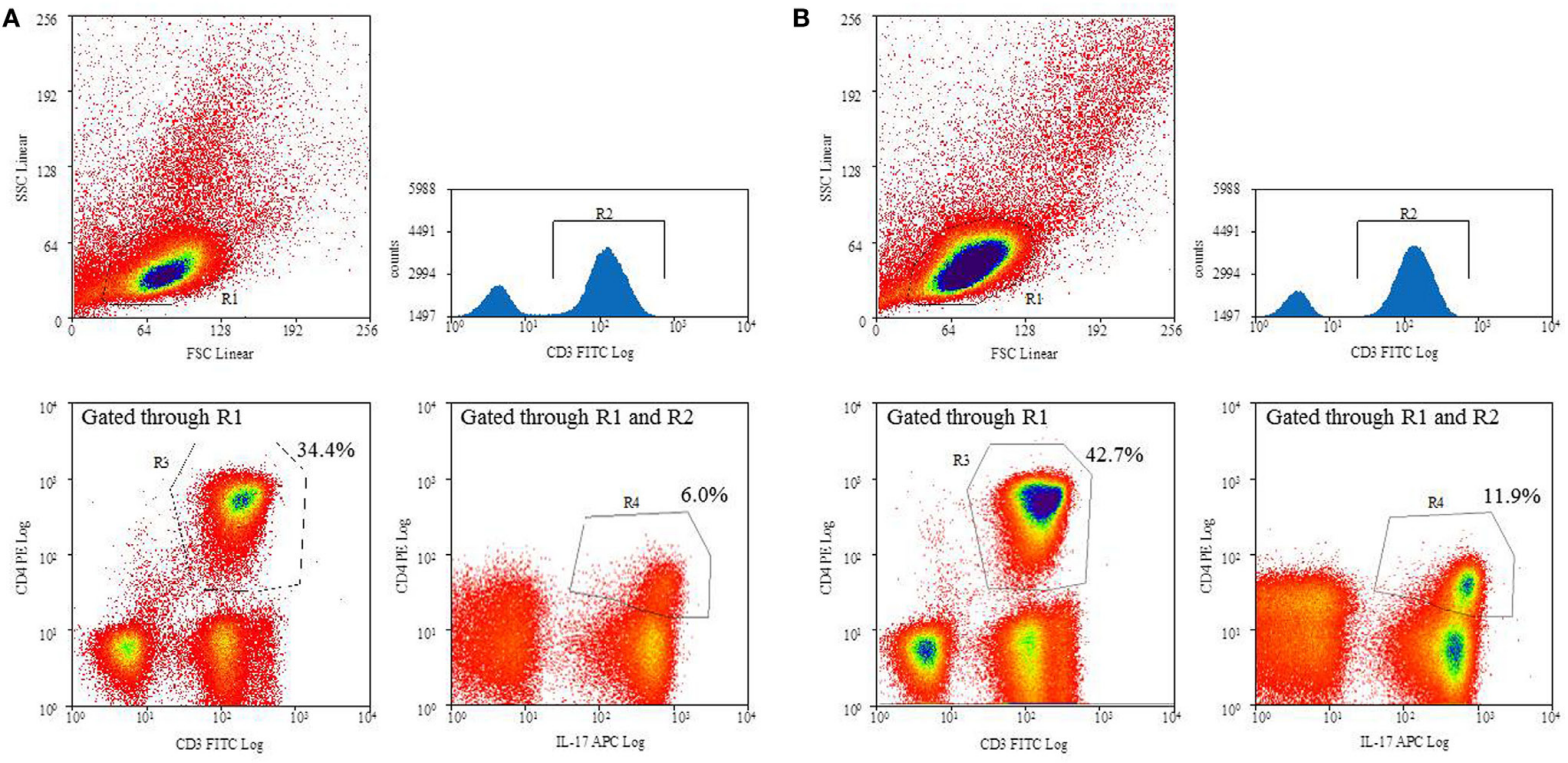
Definition of T_H_17 cells: peripheral blood mononuclear cells were stimulated with Ionomycin *in vitro* followed by staining with anti-CD3 (FITC), anti-CD4 (PE), and anti-IL-17A (APC). Representative flow cytometry analyses of a normal weight **(A)** and overweight **(B)** child, respectively.

Flow cytometry was performed on a 4-color FacsCalibur flow cytometer (BD Biosciences) using summit 4.3 software (Beckman Coulter, Krefeld, Germany). Gates were preset and the measurements were performed blinded for sample identity. T_H_17 cells were defined as CD3^+^CD4^+^IL-17A^ic+^ (22) and were evaluated as percentage of T_H_ lymphocytes (CD3^+^CD4^+^) (Figure 1).

### RNA Isolation, cDNA Transcription, and Real-time PCR

An aliquot of the stimulated PBMCs was used for messenger RNA (mRNA) isolation with TriFast (peqlab, Erlangen, Germany) and subsequent DNase I digestion (Desoxyribonuclease I, Amplification Grade, invitrogen, Darmstadt, Germany), following the manufacturer’s instructions, respectively. cDNA was synthesized from 2 µl of mRNA using Omniscript R Kit (QIAGEN, Hilden) and Oligo(dT)_18_ primer and RiboLock RNase Inhibitor (Thermo Fisher Scientific, Nidderau, Germany). cDNA concentration was measured on a NanoDrop 2000c (Thermo Fisher Scientific, Nidderau, Germany) and was diluted by addition of RNase free water (Water, Mol Bio grade DNase-, RNase-, and Protease-free; 5 PRIME, Hamburg, Germany) into a concentration of 1,000 ng/µl.

Real-time PCR was performed on each cDNA sample as duplicate to determine relative expression of RORC- and IL-17A-mRNA and was normalized by Elongation factor 1-α (EF-1-α) as a housekeeping gene and calculated by the 2^−ΔΔCt^ formula. EF-1-α was used because its expression in T cell culture is more stable than other housekeeping genes such as glyceraldehyde 3-phosphate dehydrogenase (23). 4.7 µl fluorochrom (SsoAdvanced SYBR Green Supermix; Biorad Laboratories, Munich, Germany), 4.2 µl RNase free water (Water, Mol Bio grade DNase-, RNase-, and Protease-free; 5 PRIME, Hamburg, Germany), 0.1 µl primer mix (all eurofins MWG Synthesis, Ebersberg, Germany) and 1 µl template were used per well of a 96-well plate. The following primers were used for real-time PCRs:

**Table:** 

EF-1-α-primer:	5′-CTGAACCATCCAGGCCAAAT-3′; 5′-GCCGTGTGGCAATCCAAT-3′
RORC-primer:	5′-GCCTTTCATCATCATCTCTGC-3′; 5′-GAAGATCTGCAGCCTTTCCA-3′
IL-17A-primer:	5′-CCCCAGTTGATTGGAAGAAA-3′; 5′-TTCGTGGGATTGTGATTCCT-3′.

An iQ5 (iQ5 Multicolor Real Time PCR Detector System; Biorad Laboratories, Munich, Germany) was used as thermocycler for real-time PCR. Real-time PCR analyses were executed with Bio-rad iQ5 Standard Edition software (Biorad Laboratories, Munich, Germany).

### Statistical Analysis

Statistical analyses were performed using Prism 5.0 (GraphPad Software, La Jolla, CA, USA). Group differences were tested using either the two-tailed Student’s *t*-test or, in case of non-normally distributed data, the Mann–Whitney *U* test. Violation of the normality assumption was assessed by Kolmogorov–Smirnov test. Analogously, Pearson and Spearman correlation was calculated for surface and intracellular staining for normally or non-normally distributed data, respectively. Differences with *p*-values of *p* < 0.05 were deemed significant.

## Results

### Subjects

65 children between 4 and 17 years of age were enrolled in our study—15 were overweight and 50 were not overweight as controls. The overweight children had a mean BMI-percentile of 97.12 ± 2.68 in comparison to 39.74 ± 27.04 in the control group. The patients’ anthropometric data are shown in Table 1.

**Table 1:** Characteristics of the study subjects.

Study group	Overweight	Controls	*p*
Number	15	50	
Female No. (%)	8 (53%)	29 (58%)	n.s.
Age (mean, SD) [years]	11.96 (3.21)	10.18 (3.15)	n.s.
Height (mean, SD) [cm]	150.39 (15.83)	140.77 (18.75)	n.s.
Weight (mean, SD) [kg]	60.00 (20.52)	33.45 (12.41)	*p* < 0.0001
Body mass index (BMI) (mean, SD) [kg/m^2^]	26.75 (4.08)	16.73 (2.39)	*p* < 0.0001
BMI-percentile (mean, SD) [%]	97.12 (2.68)	39.74 (27.04)	*p* < 0.0001
Leukocytes (mean, SD) [G/l] cells = (mean, SD) [G/l]	7.13 (1.41)	5.71 (1.82)	n.s.

*n.s., not significant*.

According to the inclusion criteria, all patients had a negative history of asthma, atopic dermatitis, allergic rhinoconjunctivitis, and autoimmune diseases. None of the children had an acute infection during the 2 weeks prior to the study enrollment as confirmed by medical history. A current infection was excluded by physical examination and measurement of C-reactive protein.

### T_H_17 Cell Frequency Is Elevated in Overweight Children

**Figure 2:**
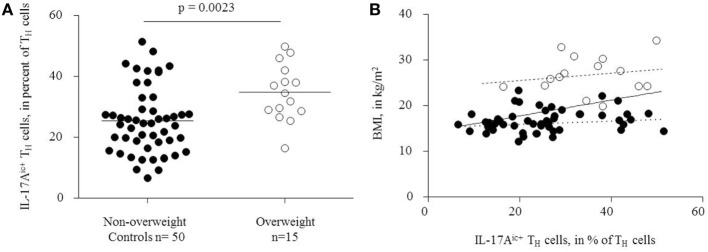
**(A)** Overweight children had significantly higher T_H_17 cell frequencies, defined as IL-17A^ic+^ T_H_ cells, than controls. **(B)** Positive correlation between circulating T_H_17 cells and body mass index, solid regression line (*r* = 0.42, *p* = 0.0005). Filled dots: non-overweight controls, dotted regression line (*r* = 0.17, *p* = 0.27). Empty dots: overweight children, dashed regression line (*r* = 0.27, *p* = 0.33).

Flow cytometry analysis showed significantly higher T_H_17 cell frequencies after stimulation in overweight children than in non-overweight controls [34.7 ± 1.54% (mean ± SEM), and 25.4 ± 2.38%], respectively (*p* = 0.0023; Figure 2A).

T_H_17 cell frequencies correlated positively with the absolute BMI (*r* = 0.42, *p* = 0.0005, Figure 2B). In contrast, IL-17 expression was similar among CD3^+^CD4^−^ cells from non-overweight controls (45.9 ± 16.0) and overweight children (50.1 ± 17.3%) (*p* = 0.384, data not shown). Thus, we did not find signs of an unspecific stimulation of IL-17 production in non-T_H_17 cells. Separate analyses of the overweight and non-overweight groups yielded no significant correlation between BMI and T_H_17 cell frequencies (*r* = 0.27, *p* = 0.33; and *r* = 0.17, *p* = 0.23, respectively, Figure 2B).

### RORC- and IL-17A Expression Is Elevated in Overweight Children

**Figure 3:**
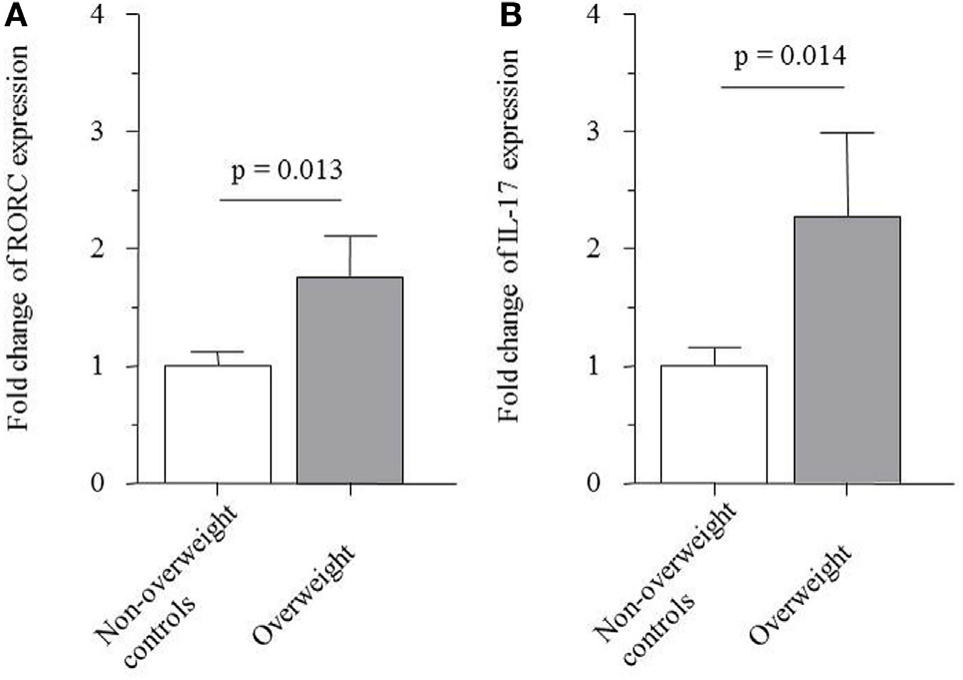
Messenger RNA expression of **(A)** transcription factor receptor-related orphan receptor C (RORC) (1.76 ± 0.35 versus 1.00 ± 0.13; *p* = 0.013) and **(B)** IL-17A (2.27 ± 0.72 versus 1.00 ± 0.16; *p* = 0.014) by PBMCs from non-overweight controls and overweight patients.

Real-time PCR revealed that the relative mRNA expression of the transcription factor RORC and of IL-17A, which are both associated with the T_H_17 cell lineage, were 1.8- and 2.3-fold elevated in stimulated PBMCs from overweight children compared to non-overweight children, respectively (1.76 ± 0.35 versus 1.00 ± 0.13; *p* = 0.013; Figure 3A and 2.27 ± 0.72 versus 1.00 ± 0.16; *p* = 0.014; Figure 3B).

## Discussion

In this study, we observed that in the absence of acute or chronic inflammatory diseases, the frequency of circulating T_H_17 cells was significantly increased in overweight children compared to non-overweight controls and there was a positive correlation between T_H_17 cell frequency and BMI. In agreement with this, we registered a significantly higher expression of RORC- and IL-17A-mRNA transcripts after stimulation in PBMCs from overweight children.

Overweight and chronic inflammatory diseases are imprinted during the fetal and early postnatal periods, and the metabolic and inflammatory phenotypes of these diseases occur during the dynamic development of the immune system in children and juveniles (24). It is known from other studies that biomarkers associated with overweight may differ significantly between obese juveniles and adults [e.g., Ref. (25)]. This demonstrates that findings on metabolic and inflammatory diseases cannot be extrapolated from adults to children without restrictions. Thus, we chose to study the frequency of T_H_17 cells in children and juveniles before the metabolism and the immune system reach maturity.

Łuczyński et al. recently reported elevated T_H_17 cells in peripheral blood of children with central obesity and in children with diabetes mellitus I (19). Since obesity is linked with an increased risk for allergic and autoimmune diseases and T_H_17 cells were increased in patients with such conditions, we studied obese individuals without concomitant allergies and autoimmune diseases (5, 6, 8–10, 22).

The role of T_H_17 cells in the development of allergies is still under investigation. Probably, various pro-inflammatory and anti-inflammatory subpopulations of T_H_17 cells play unique roles in orchestrating the allergic inflammation (26). The cytokine production of T_H_17 cells appears to be more dynamic than previously anticipated depending on the signals provided by the microenvironment (26). Compared to healthy controls, IL-17A expression was higher in cultured T-lymphocytes from patients with mild–moderate asthma and persistent allergic rhinitis and decreased after anti-inflammatory therapy with inhalative corticosteroids (22). IL-17A influences the acute inflammatory response by upregulating IL-8 secretion in airway epithelial cells and also initiates airway remodeling by affecting the airway smooth muscle cells (27).

Since BMI standard values vary throughout childhood and between various ethnicities, we used the German percentiles that were obtained from measurements of 34,422 children (21). Due to the great variance of T_H_17 cell frequencies, they overlap between the overweight and non-overweight groups (Figure 2A). Intriguingly, BMI and T_H_17 cell frequency apparently do not show a linear correlation, since we found no significant correlation between BMI and T_H_17 cells when analyzing overweight and non-overweight children separately. This could indicate that the T_H_17 bias develops above a threshold BMI that provides a pro T_H_17 inflammatory environment or that the sample sizes were too small within the subgroups.

One limitation of the study is that due to the limited cell numbers in the pediatric samples, the expression of RORC and IL-17A transcripts were investigated in unsorted PBMCs. However, multiple previous studies have confirmed that CD4^+^ T cells are the major (although not exclusive) source of IL-17 and RORC expression in similar experimental settings as ours [e.g., Ref. (8, 22)]. In accord with this, we found that the number of IL-17^ic+^ cells did not increase among CD3^+^CD4^−^ cells but only among CD3^+^CD4^+^ cells after *in vitro* stimulation (data not shown).

T_H_ cell subsets are defined by characteristic expression patterns of surface antigens, cytokines, and master transcription factors. For instance, human T_H_1 cells produce interferon-γ and Tbet (28), T_H_2 cells produce IL-4 and GATA3 (29, 30), and T_H_17 cells produce IL-17A and RORC, which is orthologous to retinoic acid receptor-related orphan receptor γt (RORγt) in mice (8, 31). RORC as a master transcription factor is necessary for the differentiation of naïve T_H_ cells into T_H_17 cells and is important for the production of T_H_17 cell effector cytokines in humans (32, 33).

In murine models, RORγt could induce IL-17A production *in vitro* (31). RORγt- and RORα-loss results in a total T_H_17 deficiency, single RORγt-loss in a relative one. In human T_H_17 cells, similar effects were observed: RORC-inhibition led to a reduced IL-17A-production and RORC-overexpression to an elevated differentiation into T_H_17 cells (34).

As effector memory T_H_17 cells, they secrete IL-21, IL-22, and IL-26, and express CCL20, CCR6, and CD161 (12, 35, 36). Multiple different staining protocols have been deduced from these lineage specific characteristics, but the complexity and insufficient understanding of the T_H_17 cell subsets precluded the development of a universally accepted standard definition. In this study, the staining protocol defines T_H_17 cells as CD3^+^CD4^+^IL-17A^ic+^ lymphocytes after stimulation—which is a common definition of flow cytometric assessed T_H_17 cells—with the strong, but unspecific stimulus Ionomycin (22). Possibly, a fraction of the CD3^+^CD4^+^IL-17A^ic+^ cells might not be classical T_H_17 cells but NKT cells, some of which can be CD4^+^ and may have the potential to express IL-17; however, according to previous work, this fraction is probably very small (37). It is unlikely that this short time culture has a polarizing effect toward the development of T_H_17 cells but it stimulates IL-17A expression in those cells that have already undergone polarization (38). According to previous studies with similar protocols, it is also unlikely that non-IL-17A-expressing T_H_ cells vanish more quickly than their IL-17A-positive counterparts (38). Probably, less T_H_17 cells are actively producing IL-17A *in vivo* than *in vitro* following stimulation, since T_H_17 cells are characterized by their ability to produce IL-17A and not by actively producing it continuously.

Obesity does not only interfere with metabolic, but also with immunological pathways and is associated with low-grade chronic inflammation (39). Several groups reported an association between obesity and T_H_17 cells in mouse models. Winer et al. induced obesity by high fat diet and measured elevated T_H_17 cells and IL-17A production in murine spleens (17), and Chen et al. detected more T_H_17 cells in adipose tissue of obese mice compared to normal weight controls (18). Furthermore, T_H_17 cells were elevated in a model of obese mice that developed inflammatory arthritis. Thus, T_H_17 cells might contribute to the pathogenesis of inflammatory arthritis in obese mice (40). Some studies found elevated frequencies of T_H_17 cells or their cytokines in children with type 1 diabetes mellitus (41, 42). IL-17A secretion by human T_H_17 cells was enhanced in the presence of adipose-derived stem cells *in vitro* (43). Dendritic cells, sorted from subcutaneous adipose tissue of obese patients, can induce T_H_17 cell differentiation *in vitro* (44). Interestingly, IL-17A gene expression in CD3^+^ cells from subcutaneous adipose tissue was elevated overweight patients compared to normal weight patients (44). In lean state, M2 macrophages, T_reg_ cells, T_H_2 cells, and NKT cells predominate, while M1 macrophages, T_H_1 cells, B cells, mast cells, and neutrophils predominate in obese individuals (6, 39). Adipose tissue produces adipokines which predominantly have a pro-inflammatory effect. Some of the identified adipokines include IL-6, IL-8, TNF-α, and IL-18 (39). TNF-α and IL-6 are required for T_H_17 cell differentiation (45). This pathway potentially leads to an increase of T_H_17 cell frequency in obese individuals. Leptin receptor signaling seems to be a further requirement for murine T_H_17 cell differentiation (46). The serum leptin concentration is elevated in obese individuals. Moreover, in humans, being overweight is associated with higher amounts of circulating neutrophils, which are attracted, among other things, by IL-17A (14, 47–49). In addition, elevated levels of myeloperoxidase were found in obese women, which coincide with higher levels of neutrophils as well (47).

Obesity can be a reason as well as a consequence of oxidative stress (50). One can hypothesize that oxidative stress might in part cause the association between obesity and allergies since obesity is associated with an overload of antioxidant compounds that promote a T_H_2-biased immune state as known from allergies (50). In support of this hypothesis, oxidative stress led to upregulated IL-17A serum levels in mice (51). In systemic lupus erythematosus, oxidative stress can cause mammalian target of rapamycin (mTOR) activation, and this mTOR activation in turn is suggested to stimulate T_H_17 cell differentiation (52, 53).

## Conclusion

In this study, we observed an elevated T_H_17 cell frequency as well as elevated RORC- and IL-17A-mRNA expression in peripheral blood of overweight children without allergic asthma, allergic rhinoconjunctivitis, atopic dermatitis, or autoimmune disease. To our knowledge, this is the first study on T_H_17 cells in overweight pediatric patients that excludes potentially confounding chronic inflammatory diseases other than obesity. Elevated T_H_17 cells might contribute to the pro-inflammatory state in overweight children. Further studies are needed to specify the underlying pathomechanism of this association. In conclusion, future studies on the frequency of circulating T_H_17 cells should control for the BMI.

## Availability of Data and Materials

The datasets supporting the conclusions of this article are included within the article or are available from the authors upon request.

## Author Information

Theresa Isabell Schindler, University Hospital Marburg, Medical Faculty, Philipps University Marburg, Children’s Hospital, Baldingerstraße, D-35043 Marburg, Phone: +49 6421-58 62650, Fax: +49 6421-58 68970, E-Mail: theresaschindler@gmx.de; Johanna-Josophina Wagner, HELIOS Klinikum Berlin-Buch, Department of Pediatrics, Schwanebecker Chaussee 50, D-13125 Berlin, Phone: +49 30 9401-14574, Fax: +49 309401-54509, E-Mail: johannaj.wagner@gmail.com; Sybelle Goedicke-Fritz, University Hospital Marburg, Medical Faculty, Philipps University Marburg, Children’s Hospital, Baldingerstraße, D-35043 Marburg, Phone: +49 6421-58 62650, Fax: +49 6421-58 68970, E-Mail: goedicke@med.uni-marburg.de; Tobias Rogosch, CSL Behring, Clinical Development, Emil-von-Behring-Straße 76, D-35041 Marburg, Germany, Phone: +49 6421 39-7179, Fax: +49 6421 39-865-7179, E-Mail: Tobias.Rogosch@cslbehring.com; Verena Coccejus, University Hospital Marburg, Medical Faculty, Philipps University Marburg, Children’s Hospital, Baldingerstraße, D-35043 Marburg, Phone: +49 6421-58 62650, Fax: +49 6421-58 68970, E-Mail: verenacoccejus@aol.com; Verena Laudenbach, University Hospital Marburg, Medical Faculty, Philipps University Marburg, Children’s Hospital, Baldingerstraße, D-35043 Marburg, Phone: +49 6421-58 62650, Fax: +49 6421-58 68970, E-Mail: v.laudenbach@web.de, Wilfried Nikolaizik, University Hospital Marburg, Medical Faculty, Philipps University Marburg, Children’s Hospital, Baldingerstraße, D-35043 Marburg, Phone: +49 6421-58 62650, Fax: +49 6421-58 68970, E-Mail: wilfried.nikolaizik@med.uni-marburg.de, Christoph Härtel, Department of Pediatrics, University of Luebeck, RatzeburgerAllee 160, 23538 Lübeck, Germany, Phone: +49-451-500 2685, Fax: +49-451-500 6222, E-mail: christoph.haertel@uksh.de; Rolf Felix Maier, University Hospital Marburg, Medical Faculty, Philipps University Marburg, Children’s Hospital, Baldingerstraße, D-35043 Marburg, Phone: +49 6421-58 62650, Fax: +49 6421-58 68970, E-Mail: rolf.maier@med.uni-marburg.de; Sebastian Kerzel, Department of Pediatric Pneumology and Allergy, University Children’s Hospital Regensburg (KUNO), Campus St. Hedwig, D-93049 Regensburg, Germany, Phone: +49 941 369 95817, Fax: +49 941 369 5424, E-Mail: sebastian.kerzel@ukr.de; Michael Zemlin, Department of Pediatrics and Neonatology, University Children’s Hospital of Saarland, Homburg Germany, Kirrberger Str. 100, D-66241 Homburg, Phone: +49 6841 16 28301, Fax: +49 6841 16 28310, E-Mail: michael.zemlin@uks.eu.

## Ethics Statement

This study was carried out in accordance with the recommendations of the Ethics Committee of the Philipps-University Marburg. All parents gave written informed consent in accordance with the Declaration of Helsinki. The protocol was approved by the Ethics Committee of the Philipps-University Marburg with the identifier AZ 91/11.

## Author Contributions

SK, MZ, TR, WN, J-JW, and RM designed the study. TS, VC, VL, and SG-F performed the experiments and evaluated the data. All authors contributed to writing the manuscript.

## Conflict of Interest Statement

Tobias Rogosch is an employee of CSL Behring. The remaining authors declare that they have no competing interests.

## Abbreviations

BMI, body mass index; CCR, chemokine receptor; CD, cluster of differentiation; cDNA, complementary DNA; EF-1-α, elongation factor 1-α; IL, interleukin; mRNA, messenger RNA; PBMC, peripheral blood mononuclear cell; RORγt, retinoic acid receptor-related orphan receptor γt; RORC, retinoic acid receptor-related orphan receptor C; T_H_, T helper; T_H_1, T helper 1; T_H_2, T helper 2; T_H_17, T helper 17.
